# Innovative Metrics for Reporting and Comparing the Glycan Structural Profile in Biotherapeutics

**DOI:** 10.3390/molecules28083304

**Published:** 2023-04-07

**Authors:** Renato Mastrangeli, Abhijeet Satwekar, Horst Bierau

**Affiliations:** 1 Global CMC Development Technology & Innovation, CMC Science & Intelligence, Merck Serono SpA (An affiliate of Merck KGaA, Darmstadt, Germany), Guidonia Montecelio, 00012 Rome, Italy; renato.mastrangeli@merckgroup.com; 2 Global CMC Development, Global Analytical Development, Global Analytical–Pharmaceutical Science & Innovation, Merck Serono SpA (An affiliate of Merck KGaA, Darmstadt, Germany), Guidonia Montecelio, 00012 Rome, Italy; abhijeet.satwekar@merckgroup.com

**Keywords:** biotherapeutics, N-glycosylation, glycosylation index, glycan analysis, standardization comparability, biosimilarity, glyco-similarity, characterization, glycopeptide mapping

## Abstract

Glycosylation is a critical quality attribute in biotherapeutics, impacting properties such as protein stability, solubility, clearance rate, efficacy, immunogenicity, and safety. Due to the heterogenic and complex nature of protein glycosylation, comprehensive characterization is demanding. Moreover, the lack of standardized metrics for evaluating and comparing glycosylation profiles hinders comparability studies and the establishment of manufacturing control strategies. To address both challenges, we propose a standardized approach based on novel metrics for a comprehensive glycosylation fingerprint which greatly facilitates the reporting and objective comparison of glycosylation profiles. The analytical workflow is based on a liquid chromatography–mass spectrometry-based multi-attribute method. Based on the analytical data, a matrix of glycosylation-related quality attributes, both at site-specific and whole molecule level, are computed, which provide metrics for a comprehensive product glycosylation fingerprint. Two case studies illustrate the applicability of the proposed indices as a standardized and versatile approach for reporting all dimensions of the glycosylation profile. The proposed approach further facilitates the assessments of risks associated with changes in the glycosylation profile that may affect efficacy, clearance, and immunogenicity.

## 1. Introduction

Recombinant glycoproteins (monoclonal antibodies, cytokines, hormones, and Fc-fusion molecules) are important therapeutics that are used to treat diseases such as cancer, autoimmunity, infections, inflammation, and endocrinological disorders; their use is broadening in clinical practice. Among different types of biopharmaceuticals, monoclonal antibodies are the fastest growing class of biologicals. A relatively high prevalence of hypersensitivity reactions (HSRs) to biologics is observed in patients, and although most of the underlying mechanisms still remain unclear [1,2], some HSRs can be attributed to undesired glycan structures, e.g., the xeno-antigenic glycan galactose-α1,3-galactose (αGal) present in some biotherapeutics [3,4,5,6]. Moreover, anti-drug antibody (ADA)-mediated reactions may be facilitated by glycan interactions, e.g., mannose with C-type lectin receptors present on dendritic cells [7,8], that can lead to the uptake of the biologic and to its presentation to T cells that help B cells to produce ADAs. The protein glycosylation profile can therefore profoundly affect the molecular properties of a therapeutic in terms of stability, solubility, clearance rate, efficacy, immunogenicity, and safety. 

The glycosylation profile of a given drug depends, amongst other factors, on the host cell line [9] and is very sensitive to cell culture conditions [10]. Further, it may be impacted by downstream processing activities in the biomanufacturing process [11,12]. Glycosylation is therefore considered as one of the critical quality attributes (CQAs) of glycosylated biopharmaceutical drugs [13,14] that must be closely monitored and controlled during the development and manufacturing of New Biological Entities (NBEs) and biosimilars. The glycan profile resulting from a given manufacturing process can therefore be considered a process fingerprint. To ensure safety and efficacy, regulatory agencies require a stringent glycan analysis as an essential part of quality control strategy of a manufactured biotherapeutic. Thus, a comprehensive comparison is required to demonstrate comparability, e.g., after the manufacturing process changes or for asserting similarity with the reference medicinal product. 

**Figure 1:**
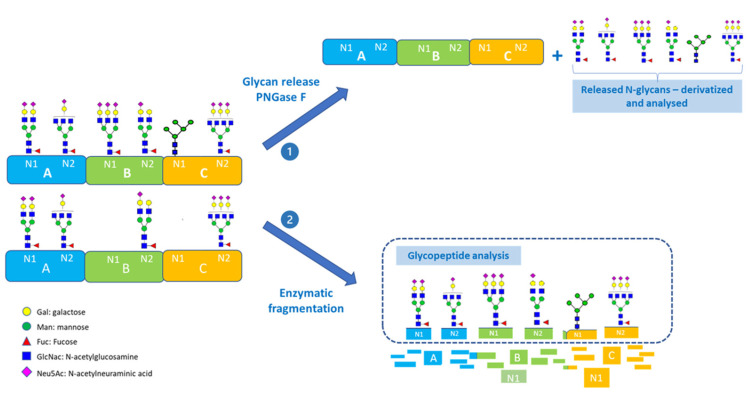
Schematic workflow exemplifying the glycan analysis of a complex glycoprotein with three domains (A, B and C) containing two N-glycosylation sites in each domain. Domains B and C are partially glycosylated at the N1 site. Glycan analysis by (1) glycan release method by PNGase F: the site-specific information is lost; or by (2) glycopeptide mapping: the site-specific information is maintained.

**Figure 2:**
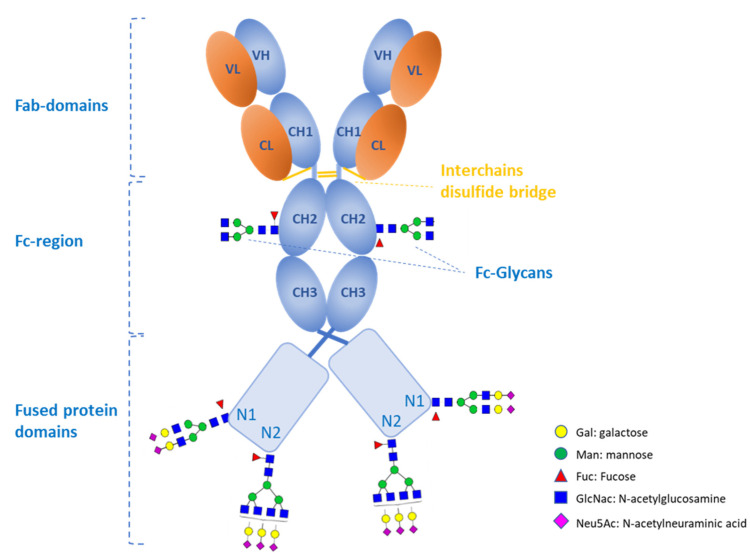
An example of an IgG1 antibody fusion molecule containing 3 N-linked glycans in each heavy chain: one in the CH2 domain of the Fc region and two (N1 and N2) in the fused protein domain. Each site is characterized by its own glycosylation profile. Abbreviations used: VL and CL—variable and constant domains, respectively, of the light chain; VH and CH—variable and constant domains, respectively, of the heavy chain; CH1, CH2, and CH3—constant domains 1, 2, and 3, respectively, of the heavy chain.

Glycan analysis is a challenging task, particularly for multi-domain proteins or proteins that contain multiple glycosylation sites. Each site can be partially or fully glycosylated with a variety of different glycoforms, adding to the complexity of the analysis. (Figure 1 and Figure 2). 

As a result, glycoproteins typically carry a high degree of glycan heterogeneity, both at the overall glycoprotein level (macro-heterogeneity) and at each individual glycosylation site (micro-heterogeneity). Different glycan types can contribute to different protein properties. Therefore, batch-to-batch consistency of the overall glycosylation profile during manufacturing must be monitored and maintained at a site-specific level. 

Complementary approaches to characterize protein glycosylation include the analysis of intact glycoproteins or glycopeptides after enzymatic digestion, and the structural analysis of enzymatically released glycans [15]. Glycan release methods are still considered the “best approaches” for the determination and characterization of the fine details of glycan structures present in a glycoprotein [16], and the enzymatic release of N-glycans is the preferred method [17]. 

With glycoproteins consisting of multiple glycosylation sites, the N-glycan release method results in the pooling of all glycans present. Therefore, site-specific glycosylation information (Figure 1) is lost, which limits the understanding of the site-specific functional impact attributable to glycosylation. In addition, results obtained for the released N-glycan pool may be confounded by the presence of glycans released from contaminating residual host cell proteins or released from noncanonical glycosylation sites, e.g., the Asn -X-Cys motif [14,18,19,20], or the Asn-X-Gly a motif present in the CH1 constant domain of IgG1 and IgG2 antibodies [21,22], where X is any amino acid except proline. Furthermore, when dealing with antibodies or fusion proteins, as shown in Figure 2, the analysis of released glycans may confound the results related to the Fc-glycan distribution which plays a pivotal role for the Fc effector functions. 

Generally, a wide panel of complementary analytical methods are employed to monitor and characterize glycosylation profiles in glycoproteins [15,16,23]. To characterize complex glycoproteins containing multiple glycosylation sites, the analytical strategy needs to deliver precise information and should ideally be high throughput in order to facilitate industrial routine use. However, glycosylation analysis is challenging due to the heterogeneity and complexity of the structures to be analyzed, e.g., N- or O-linked glycosylation, with considerable micro-heterogeneity, i.e., different substitution levels of terminal sugars such as galactose and sialic acid, as well as specific linkage types [23]. Moreover, the lack of standardized metrics [10] further complicates data evaluation and comparison with literature data. 

Blondeel and colleagues introduced five indices, expressed as percentages, to monitor the shifts in the antibody glycosylation patterns resulting from different cell culture supplementation regimes during bioprocessing: the galactosylation index (GI), the sialylation index (SI), the fucosylation index (FI), the antennarity index (AI), and the mannose index (MI). The use of indices facilitates the comparison of samples. Similar sialylation and galactosylation indices (expressed as proportions) were applied by Liu and colleagues to monitor the impact of supplementation strategies on antibody galactosylation and sialylation profiles (by glycan release) during bioprocessing [24]. Although these indices facilitate monitoring of the effectiveness of methodologies to modulate particular glycoforms, e.g., in process development or improvement, they are less useful to provide a detailed overall representation of the glycan population [10].

For other protein classes, further glycosylation-related indices such as the Z-number, defined as the hypothetical charge number, which was introduced in 1996 to easily characterize protein glycosylation [25], and the A-index (hypothetical antennarity index) [26] were used to characterize the overall released glycans in gonadotrophins. It is noteworthy to mention that the glycosylation index Z-number is still required by European Pharmacopoeia in the monograph of follitropin concentrated solution, with different Z-number ranges for follitropin-released glycans depending on the analytical method used. 

The Z-number, as described in the monograph, is calculated on released glycans and therefore does not take the site occupancy and site-specific glycan distributions into account. A limitation to using only the Z-number, is that no information on the presence of glycan-related CQAs such as high-mannose glycans or Neu5Gc is provided. Moreover, Z-numbers can be ambiguous as it is possible to obtain identical Z-numbers with significantly different glycan distributions and site occupancies. Thus, the Z-number obtained after the glycan release does not fully represent the actual glycan distribution and may not be a suitable indicator for glyco-similarity between different products or versus a reference product.

Due to the limitations of the reported indices, a harmonized, universally applicable, and adaptable matrix of glycan-specific indices with an overall aim to facilitate the reporting and representation, along with the visualization and interpretation of the protein glycosylation content of a glycoprotein product is proposed. The suggested matrix can be readily adapted based upon the employed expression host, the molecular characteristics, as well as the mode of action of the glycoprotein product (Table 1). 

These indices intuitively represent and characterize the glycan distribution in accordance with their structural attributes and can be calculated for each site/subunit/domain or at the whole molecule level. The proposed comparative matrix can therefore provide a fingerprint of relevant glycan-related CQAs resulting from a specific manufacturing process for a given biotherapeutic class. The matrix can be adapted for a given product, e.g., by selecting a reduced subset of indices to cover the most relevant glycan-related CQAs for a given class of protein therapeutic. The current study and description of the matrix focusses only on N-linked oligosaccharides, even though the matrix can also be applied to O-linked oligosaccharides.

## 2. Main N-Glycan-Related CQAs and Indices

Biotherapeutics can be produced using different expression systems which differ with regards to their enzymatic glycosylation machinery. Based on the selected expression system, specific post-translational modifications (PTMs) in terms of monosaccharide composition of glycans and monosaccharide linkage types, as well as their levels, can be found (Table 2). 

Depending on the degree of intracellular glycan processing, a heterogeneous N-linked glycan distribution arises comprising varying amounts of high mannose, hybrid- and complex-type glycans. The choice of the expression system and the manufacturing process conditions are therefore important parameters defining the most relevant glycan-related CQA indices to be determined at a site-specific level, and at an overall molecule level (Table 1).

The following sections report the main glycan-related indices/CQAs to be considered to build the characterization matrix of therapeutic glycoproteins.

### 2.1. Galactose-α1,3-Galactose (αGal)

The presence of αGal in biotherapeutics is one of the main concerns regarding the potential impact on safety, immunogenicity, bioavailability, and efficacy [4,5,28,29,30,31]. αGal is a xeno-antigenic glycan structure that is not synthetized in higher primates due to an inactivating mutation of the α1,3-galactosyltransferase gene [32]. Upon exposure to this epitope, humans produce large amounts of anti-αGal-specific antibodies. αGal is well known to be the major xeno-antigen stimulating the human immune response. The presence of this epitope represents a major barrier to xeno-transplantation [33] and is a significant factor involved in structural valve deterioration of bioprosthetic heart valves [34]. Allergic reactions to αGal-containing foods are well known and IgE antibodies to αGal can elicit serious reactions [6,31]. Preexisting circulating anti-αGal IgE antibodies in humans may therefore impact the safety and the bioavailability profile of a biotherapeutic. However, anti-αGal IgE does not significantly interact with the αGal epitope when αGal is present in the Fc domain of mAbs [35]. 

The αGal index (αGI) represents the number of αGal units present at a given glycosylation site or on the whole molecule level. Each antenna of a glycan, at least theoretically, can be terminated by αGal. Hence, αGI can theoretically range from 0 to AI (the number of antennae present). This index is relevant when murine cell lines, such as Sp2/0 and NS0, expressing the α1,3-galactosyltransferase gene [36,37] are employed for biotherapeutics manufacturing. In Chinese hamster ovary (CHO) cells this gene was found inactivated [38]. Nevertheless, low amounts of the αGal epitope have been found in proteins recombinantly expressed in CHO which was attributed to N-acetyllactosaminide 3-α-galactosyltransferase-1 activity, e.g., in the fusion molecule abatacept (CTLA4-IgG) where αGal was observed in around 0.2% of total glycans [37]. In CHO, it is presumed that the presence of the αGal epitope arises during clonal selection due to differential expression of this gene in different sub-clonal populations [37]. The potential levels of αGal in CHO are expected to be very low in comparison to levels commonly found in murine cell lines; nevertheless, it is important to monitor and control the potential presence of this epitope in CHO, especially during the phase of clone selection. 

In terms of risk, αGal is less of a concern when present in the Fc region, both due to the low content and the masking activity of the Fc region [35]. Meanwhile, a higher risk is expected when the αGal is exposed to the environment. The αGal index is thus directly correlated with the safety, immunogenicity, and clearance risk.

### 2.2. N-Glycolylneuraminic Acids (Neu5Gc) 

Neu5Gc is a xeno-autoantigenic glycan, commonly found as a dietary non-human sugar that is metabolized by human cells and incorporated in glycan structures, that are exposed on the cell surface. As xeno-autoantigen, the resulting Neu5Gc-containing glycans potentially induce an immune response. Although Neu5Gc has a lower antigenicity compared with the αGal xeno-antigen [33,39], the incorporation of Neu5Gc in therapeutic recombinant proteins raises clinical concerns due to its immunogenic potential and the high prevalence of pre-existing anti-Neu5Gc antibodies in humans that can also modulate the clearance of the biotherapeutic [40,41]. Anti-Neu5Gc antibodies are generated during early infancy and persist during adulthood at a level of approximately 0.1–0.2% of circulating immunoglobulins. These antibodies can also be the cause of the rejection of animal-derived transplanted organs [33,42] and animal-derived implanted biodevices, such as bioprosthetic heart valves [34]. Further considerations regarding structural and functional aspects of Neu5Gc-containing glycans and their potential impact on drug clearance, their recognition by pre-existing antibodies, and the assessment of potential risks associated with Neu5Gc containing biotherapeutics have recently been reviewed [41,43]. 

From a practical point of view, the Neu5Gc-related risk is much lower for Fc-glycans compared with that for glycans in other domains. Especially, if the Fc region carries only one Neu5Gc molecule. Increasing the number of Neu5Gc molecules (≥2) in the Fc region can increase the risk due to Neu5Gc exposure to pre-existing antibodies [44].

The proposed SI_Neu5Gc_ represents the number of Neu5Gc molecules at a given glycosylation site or at the whole molecule level. Each antenna of a glycan (at least theoretically) can be capped by Neu5Gc. Therefore, SI_Neu5Gc_ can theoretically range from 0 to AI (the number of antennae present). A lower Neu5Gc-related risk is expected for Fc glycans due to partial shielding. However, this risk is increased when the Fc region contains ≥ 2 Neu5Gc molecules. Generally, when glycans are exposed to the environment, e.g., for domains other than Fc, they are prone to be recognized by pre-existing antibodies [43,44]. The SI_Neu5Gc_ index, therefore, correlates with the immunogenicity and clearance risk [40,41,43,44].

### 2.3. High Mannose (HM) Glycoforms

HM glycoforms can be considered incompletely processed N-glycans (M_9-5_) that generally accumulate during later phases in cell culture processes. A common observation during process intensification is the correlation of HM level with increased titer, cell-specific productivity, and viable-cell density [45,46]. Pushing producer cell lines towards higher productivities can create metabolic bottlenecks resulting in limitations with regards to substrates and/or cofactors or suboptimal levels and conditions for enzymes involved in the glycosylation pathway. These factors result in the accumulation of immature glycans such as HM forms [7].

HM_9-5_ glycans do not contain fucose, and thus, mAbs containing such glycans have an increased interaction with the Fc gamma receptor III-A (FcγRIIIa) [47] responsible for triggering antibody-dependent cell cytotoxicity (ADCC) [48,49,50]. Therefore, compared with mAbs containing fucosylated glycans, HM-containing mAbs have enhanced ADCC activity [50,51]; although not at the level of mAbs containing afucosylated and galactosylated complex-type glycans [11]. In general, HM glycans are known to interact with C-type lectin receptors (DC-SIGN, Dectin-2, mannose receptor) [7,52,53]. Mannose receptors, expressed on macrophages and dendritic cells, bind glycans containing exposed mannose residues. Ligand uptake is continuous, due to receptor recycling, allowing the intracellular processing of large quantities of ligands [8]. Therefore, the presence of high mannose glycans may potentially alter function, e.g., ADCC, increase clearance [54], and likely affect immunogenicity.

The proposed index (MI) represents the number of HM glycans per site, ranging 0 ≤ MI ≤ 1, or present in the overall molecule. Hybrid structures may also partially contribute to MI. The index directly correlates with the clearance and the immunogenicity risk.

### 2.4. Hybrid Type

Hybrids are intermediated glycans generated during the conversion processing of high mannose glycans to complex-type glycans (which may or may not contain a core-fucose residue). As suggested by the name, hybrid glycans contain both terminal mannose residues at the a1,6 arm and complex-type residues at the a1,3 arm. Therefore, similar to HM glycans, the mannose residues on the a1,6 arm may result in accelerated clearance and immunogenicity. The a1,3 arm, similar to complex-type glycans, can contain one or two antennae that can be involved in clearance if not capped with sialic acid. Generally, hybrid-type N-glycans are present at very low levels and the hybrid index (HI) can therefore be exploited for process fingerprinting. 

### 2.5. Antennarity

Complex-type N-glycans are produced as a heterogeneous mixture of branched glycans. The number of branches or antennae is dependent on the action of several N-acetylglucosamine transferases [55] and generally ranges from 2 to 5. The higher the number of antennae the higher the steric hindrance of the N-glycan on the protein surface with a potential impact on protein–protein interactions. Moreover, the heterogeneity, i.e., in terms of possible glycan structures attached, increases with the degree of antennarity. 

The proposed antennarity index represents the ponderal average number of antennae (branching) present at a given glycosylation site with contributions of both complex-type (possible range 2–5) and hybrid-type glycans (possible range 1–2); HM glycans are considered as having 0 antennae. Therefore, the index has a range of 0 ≤ AI ≤ 5. For Fc-glycans, the index has a range of 0 ≤ AI ≤ 2, as the Fc-complex-type glycans are only biantennary. 

AI is therefore representative of the average glycan branching and may be relevant when antennarity is known to have an impact on the biological activity of a protein [56]. At the whole protein level, the AI can be used for process fingerprinting. In addition, it is used in the calculation of the overall sialylation extent SE, known to impact the clearance of a protein. 

### 2.6. Sialylation 

Sialylation plays a pivotal role in improving the properties of recombinant proteins in terms of solubility, biological activity, thermal stability, and circulatory half-life. In vivo, sialic acids extend the glycoprotein half-life by masking the galactose residues from asialoglycoprotein receptor (ASGPR). Compared to α2,6-linked sialic acid, its α2,3 counterpart provides a more pronounced masking effect versus ASGPR [43]. Accelerated clearance of proteins containing α2,6-linked sialic acid has been reported in rodents [27]. However, a comparable effect for human ASGPR is still to be demonstrated [43]. 

The sialylation index (SI) and the sialylation extent (SE) together describe the sialylation profile of a molecule. The SI represents the ponderal average number of sialic acid molecules (Neu5Ac, Neu5Gc, or O-acetylated sialic acids) present at a given glycosylation site or at the whole molecule level and has a range of 0 ≤ SI ≤ AI. For sialylated molecules it may further be useful to calculate the respective percentages of the Neu5Ac and Neu5Gc content (with respect to the overall number of sialic acid residues present), e.g., %_Neu5Gc_ = SI_Neu5G_/SI × 100 and %_Neu5Ac_ = SI_Neu5Ac_/SI × 100. Since commonly not all antennae are capped with sialic acid, the SE describes the ratio of the actual vs. the maximum possible number of sialic acids present (range 0 ≤ SE ≤ 1). The maximum theoretical values, SI = AI and SE = 1, can only be obtained when all antennae are fully capped with sialic acid. 

The overall charge distribution of a glycoprotein correlates with the number of sialic acid residues present. Moreover, the extent of sialylation is inversely correlated with both the clearance mediated by the ASGPR and with nonspecific binding interactions of proteins on negatively charged cell membranes [57].

For example, glycoproteins such as gonadotrophins and erythropoietin are highly glycosylated molecules resulting in a distribution of charge isoforms whose distributions correlate with the number of sialic acid residues present. A higher sialic acid content, resulting in a more acidic pI, has been reported to increase the in vivo potency due to a prolonged serum half-life [58,59,60,61]. 

The SI calculated at overall molecule level can be exploited for process fingerprinting. Moreover, this index is of particular value for the monitoring of the potential impact of glycoengineering, e.g., the addition of glycosylation sites with the aim of increasing potency or half-life [59,61]. In this case, the increased overall SI could be directly correlated with the enhanced in vivo potency.

### 2.7. Sialic Acid O-Acetylation 

O-acetylation is one of the most common PTMs that occur on sialic acid at either the 4-, 7-, 8-, or 9-position. This modification reduces the hydrophilicity of the sialic acid residue and may cause conformational changes in glycoproteins [62]. The key enzyme responsible for O-acetylation is sialate-O-acetyltransferase located in the Golgi apparatus [63]. This modification is usually labile under alkaline reaction conditions [62] and does not pose a risk in terms of immunogenicity being a natural PTM in humans. O-acetylation has been reported to inhibit the activity of mammalian sialidases [64]. The protective function versus sialidase activity may reflect a potential impact on pharmacokinetics (PK). Although, no significant effect on efficacy and PK has been reported, e.g., by increasing O-acetylation from 49% to about 71% in the biotherapeutic darbepoetin alfa [65].

The proposed index percent O-acetylated represents the % of O-acetylated sialic acid over the total sialic acid present at each site and can be exploited for process fingerprinting.

### 2.8. Bisecting N-Acetylglucosamine (Bisecting GlcNac) 

Hybrid and complex N-glycans may carry a bisecting N-acetylglucosamine (GlcNAc) group that is β1,4-linked to the core β-mannose residue [66]. This bisecting structure is not considered an antenna since it cannot be further extended. The enzyme responsible for generating the bisecting structure is the β1,4-mannosyl-glycoprotein 4-β-N-acetylglucosaminyltransferase (GlcNAc-T III, mgat3). This modification confers differential recognition properties involved in different biological processes [66]. The expression of GlcNAc-T III is correlated with a decrease in multiple branched N-glycan structures, resulting in a diverse balance among different types of N-glycans. Instead, when bisecting GlcNAc is present on Fc-glycans, it enhances ADCC activity since this modification prevents the addition of core fucose to Fc-N-glycans. For example, the Roche GlycoMAb^®^ technology is based on engineered cells overexpressing β1,4-N-acetylglucosaminyltransferase III. This platform therefore produces antibodies highly enriched in bisected, non-fucosylated N-glycans with enhanced ADCC; an example is obinutuzumab (Gazyva^TM^) an anti-CD20 commercial antibody used for the treatment of chronic lymphocytic leukemia [67]. 

The presence of bisecting GlcNAc in exposed glycans may confer specific binding properties, e.g., with the C-type lectin receptor dendritic cell inhibitory receptor 2 [68]. However, no safety issues are expected as this is a common PTM in human proteins. In contrast, potential impacts on the properties of a biotherapeutic cannot be excluded and therefore any changes in the observed levels should be evaluated.

Human cell lines can produce bisecting GlcNAc glycans while mouse cells (Sp2/0 and NS0) and CHO cells (unless engineered) have no detectable GlcNAc-TIII activity [69,70,71]. The bisecting GlcNac index (BI) represents the proportion of bisecting GlcNAc present in each site with a range of 0 ≤ BI ≤ 1. This index can be exploited for process fingerprinting. 

### 2.9. N-Acetyllactosamine (LacNAc)

This modification refers to the addition of one or more units of N-acetyllactosamine, a key structural unit in milk oligosaccharides [72], to the terminal galactose of N-glycans. LacNAc is a potential backbone for additional modifications by fucosyltransferases, sialyltransferases, and sulfotransferases. Poly-N-acetyllactosamine chains can be synthesized by repeated alternating additions of N-acetylglucosamine (GlcNAc) and galactose (Gal), catalyzed by β-1,3-N-acetylglucosaminyltransferases, and β-1,4-galactosyltransferases [73]. 

The LacNAc modification extends the N-glycan structure by adding at least two residues (GlcNAc and Gal). Although, in the case of darbepoetin alfa, N-lactosamine levels of up to 25% have been reported not to affect efficacy and PK [65]. It may be speculated that elevated levels of this modification could modulate the half-life of biotherapeutics by different mechanisms. On one hand, extending the glycan structure could increase the protein hydrodynamic radius, resulting in reduced clearance if glomerular filtration contributes to the PK of a given therapeutic, e.g., as reported for erythropoietin [74]. On the other hand, exposing more accessible Gal residues could lead to enhanced interaction with the ASGPR, when uncapped by sialic acid, leading to an increased clearance. 

N-acetyllactosamine units share affinity for galectin-3, a carbohydrate binding protein involved in protein trafficking and regulation of cellular processes [75]. Moreover, N-acetyllactosamine is the backbone for the generation of fucosylated Lewis-type II antigens such as Lewis^x^, sialyl Lewis^x^ and Lewis^y^ [76]. Therefore, potential implications of the presence of LacNAc in biotherapeutics warrant further investigation.

The proposed LacNAc index (LI) represents the number of LacNAc units present in each glycosylation site or on the whole molecule level. This index can be exploited for process fingerprinting or when LacNac is suspected to impact molecular properties. 

### 2.10. Fucosylation 

#### 2.10.1. Core α1,6 Fucosylation 

In mAbs, core fucosylation is an important quality attribute with regards to Fc effector functions. Afucosylation, i.e., the absence of core fucose, boosts the ADCC activity due to significantly improved binding to the FcγRIIIa for both 158V and 158F polymorphisms [77], while complement-dependent cytotoxicity (CDC), FcRn, FcγRI, FcγRIIa, and antigen binding remain mainly unaffected [50]. 

The relevance of fucosylation in other classes of biologics is exemplified by FSH, a heterodimeric hormone with differential core fucosylation in the glycans of its subunits. While in pituitary FSH the α-subunit glycans are almost entirely afucosylated, the β-subunit glycans carry core fucose [78], a difference which is likely due to the fact that α-subunit glycans are buried within the dimeric structure [79] and therefore less accessible for the α-1,6-fucosyltransferase enzyme responsible for core fucosylation. Although little is known about the role of FSH core-fucosylation, it can be assumed that it may affect molecular flexibility through protein-backbone interactions [80]. Consequently, it is desirable for manufacturing processes of recombinant FSH to closely mimic the differential glycosylation profile of the two subunits. 

The proposed index (cFI) represents the fraction of core-fucosylation at a given glycosylation site, with a range of 0 ≤ cFI ≤ 1. For Fc-glycans, the cFI is thus inversely correlated with ADCC. 

#### 2.10.2. Antennae Fucosylation

Fucosylation can occur also on the antennae of the N-linked glycans with the generation of Lewis-type antigens such as Lewis^x^ and sialyl Lewis^x^ as reported in CHO mutants [81]. Sialyl Lewis^X^ is a member of blood carbohydrates present on the surface of different immune cells; it is a ligand for selectins and play an important role in different physiological phenomena [82,83]. Moreover, antennae fucosylation is also correlated with the rapid clearance and cellular uptake mediated by the mannose receptor present on macrophages and dendritic cells [8,84]. Antennae fucosylation can therefore increase the clearance and likely immunogenicity.

The proposed index (aFI) represents the number of fucose residues present on antennae at a given glycosylation or at the whole molecule level.

### 2.11. Galactosylation 

#### 2.11.1. Non-Fc Glycans

In exposed glycans, the presence or absence of terminal galactose on the antennae will modulate the clearance. Consequently, a suitable metric to monitor this attribute is desirable with regards to process optimization and process fingerprinting. Since, at the whole molecule level, the impact on clearance due to exposed galactose (or exposed GlcNAc) is already implicit in the sialylation extent (SE), it is sufficient to consider the galactosylation index (GI) only at glycosylation site level.

#### 2.11.2. Fc-Glycans

Fc-glycans can have either zero (G0), one (G1), or two terminal galactose (G2) moieties. The Fc effector functions like the ADCC, the antibody-dependent cell-mediated phagocytosis (ADCP), and the CDC can potentially be affected by the level of galactosylation. A positive correlation of terminal galactose with CDC [50], ADCP [85], ADCC [86,87]), and anti-inflammatory properties of antibodies [88,89] has been reported.

Endogenous antibodies generally contain higher levels of galactosylated Fc-glycans, while in commercial mAbs agalactosylated (G0) glycans are the major Fc-glycoforms. Regarding the G1 and G2 Fc-glycans, the residues on the α1,6 and α1,3 arms of the biantennary Fc-glycans are characterized by differential interactions and mobility [90,91]. The α1,6 arm is more rigid due to H-bond interactions with the Fc structure and has been associated with enhanced ADCC [85]. While the α1,3 arm is more flexible, more accessible to sialyltransferase, and found to be correlated with a reduced ADCC [85]. Noteworthy, Aoyama and colleagues clearly demonstrated the differential impact on ADCC and CDC activity confirming that the enhanced CDC activity and the enhanced FcγRIIIa affinity is only due to the galactose present on the α1,6 arm of the Fc-glycans [92]. It has also been reported that galactosylated mAbs have different properties in terms of Fab orientation in comparison with G0-containing mAbs [90], and this may potentially translate in differential functional properties. 

Thus, for Fc glycans, due to the potential impact on stability and effector functions, the proposed galactosylation indices GI, G2, G1, G1_1,6_, and G0 can be exploited case-by-case, based on the molecules’ mechanism of action and CQA risk assessment, for a comprehensive product characterization to demonstrate process consistency and comparability/biosimilarity. 

### 2.12. Site Occupancy

Glycan microheterogeneity, i.e., the presence or absence of N-linked glycans on a specific glycosylation site, is an important determinant in terms of solubility, stability, function, clearance, and immunogenicity. For example, removal of Fc-glycans in mAbs ablates their Fc-effector functions [93]. Changes in glycan site occupancy can also modulate the biological activity of a protein. For example, the macro-heterogeneity within the four pituitary FSH glycosylated variants result in different in vivo biological activities due to the presence or absence of oligosaccharide chains at one or both sites of the FSH β-subunit [80,94]. 

Metrics for site occupancy are also relevant for risk assessments; for example, when glycans contain undesired sugars such as the non-human Neu5Gc or a-Gal which can pose a risk for PK, safety, or immunogenicity. Such a case is exemplified by tralokinumab, an interleukin (IL)-13 neutralizing IgG4 antibody produced in murine NS0 cells [95]. This antibody, in addition to the canonical Fc glycosylation site, contains two putatively exposed glycosylation sites in each of the Fab regions. These Fab glycans were found to be biantennary and triantennary complex-type structures fully capped with Neu5Gc. However, the site occupancy of these Fab glycans was very low (<1%), and clinical data demonstrated that ADA incidence against tralokinumab was low. Additionally, no differences in terms of hypersensitivity or anaphylactic reactions were found compared with the placebo groups indicating that the overall low Neu5Gc level in the Fab region was well tolerated without significant impact on safety [95].

The proposed site occupancy index (SOI) is a measurement for the macroheterogeneity arising from the presence or absence of glycans in individual glycosylation sites. The index (range 0 ≤ SOI ≤ 1, with SOI = 1, the site is fully glycosylated, and with SOI = 0, the site is not glycosylated) can be calculated at the individual glycosylation site level. The index therefore provides a metric for the potential impact or the individual contribution of a glycan, present in each glycosylation site, on different biotherapeutic properties. Furthermore, it could be employed to monitor process consistency, e.g., after process changes or during manufacturing. 

## 3. Analytical Workflow and Case Studies

### 3.1. Mass Spectrometry-Based Glycopeptide Analysis 

Mass spectrometry-based glycopeptide analysis is capable of providing quantitative and qualitative data on the comprehensive site-specific glycosylation information within a glycoprotein [16]. This approach preserves site-specific glycosylation information, potentially present in different domains. Furthermore, within the product Quality by Design (QbD) approach, aiming at establishing a suitable control strategy in manufacturing, the glycopeptide analysis is favorably compatible as a “Multi-Attribute Method” (MAM), which is capable of analyzing a number of quality attributes simultaneously [96]. Thus, a finely tuned liquid chromatography–mass spectrometry (LC-MS)-based multi-attribute method (MAM) analytical approach for the site-specific N-glycan characterization, screening, quantitation, and discovery was developed. The analytical results by this approach form the basis for the calculation of the site-specific indices as described in Section 6.2.3. Site occupancy can be determined by using label and label-free LC-MS approaches [97] following deglycosylation and digestion of the molecule as described in Section 6.2.2. 

### 3.2. Matrix of Glycan-Related CQAs 

The proposed methodology is generically applicable and can be easily adapted to different classes of biotherapeutics such as gonadotrophins, cytokines, mAbs, fusion molecules, etc. (two case studies are reported in the next sections). The indices that are relevant for a given therapeutic molecule are selected based on aspects, such as the class of biotherapeutic, the mode of action (which may be impacted by the glycosylation profile), structural aspects (domains and glycosylation sites), safety, immunogenicity, clearance, and also the manufacturing process (e.g., the type of expression host). Thus, for each molecule, the matrix is compiled from a specific subset of those indices that are relevant for the compound. For indices that are calculated at the whole molecule level, the total number of glycosylation sites, the number of homomultimeric chains, and the site occupancy need to be taken into consideration. As a result, the proposed matrix facilitates the visualization and interpretation of protein glycosylation and provides a useful assessment tool for the evaluation of glycan-related CQAs, the monitoring of bioprocesses, and to assess comparability or biosimilarity.

### 3.3. Case Study 1: Antibody Fusion Protein Expressed by CHO Cells

To illustrate this concept, we considered the example of an antibody-fusion construct, as shown schematically in Figure 2. The compound is produced in CHO cells and is composed of two heavy and two light chains interconnected by disulfide bridges. Each heavy chain bears the canonical Fc-glycosylation site, and its C-terminus is fused to a protein domain containing two additional N-glycosylation sites (N1 and N2). The Fc glycans can modulate antibody effector functions and must be characterized at the site-specific level. The glycosylation sites in the fused protein potentially impact the efficacy, clearance, and immunogenicity and must be characterized at site-specific, whole domain, and molecular levels. 

Based on these characteristics, the matrix shown in Table 3 could be selected, which allows the clear differentiation of the glycosylation features in the Fc domain from those in the fusion domain (N1 and N2). 

CHO cells, unless engineered, do not synthetize α2,6-linked sialic acid and have no relevant, detectable activity to form bisecting glycans. Furthermore, compared to murine and human cells, CHO cells are known to produce only low amounts of sialylated Fc-glycans (typically 0–2%) [98]. Therefore, the potential presence of any species with Neu5Gc in Fc-glycans is negligible. Moreover, some modifications such as O-acetylation, antennae fucosylation, and N-acetyllactosamine formation generally do not occur in Fc-glycans. Analytical data from the LC-MS MAM analysis, providing site-specific glycan information, was used to calculate the selected indices at site/domain level. 

Table 4 summarizes the analytical results for a reference in-house standard (RHS) of the model Fc-fusion protein. 

Some indices such as αGI, aFI, LI, and BI were not included as the corresponding glycans were not detected. The results shown in Table 4 were obtained in the frame of a robustness study comprising 21 different RHS analytical sessions. The matrix characterizes the glycosylation profile of the RHS at various levels as glycan site, domain, or whole molecule. It was observed that the main abundant Fc glycans are mostly non-sialylated, core fucosylated biantennary forms, with a minor presence of high mannose forms (approximately 2%). Furthermore, the RHS glycosylation sites (Fc, N1, and N2) differ in terms of antennarity (AI = 1.77, 2.64, and 3.42, respectively), and (as expected) in terms of their sialylation level (SI = ND, 1.73, and 2.43, respectively). The lower galactosylation and sialylation degree commonly seen in the Fc glycans reflects the incomplete glycan processing resulting from steric hindrance in the Fc domain affecting the galactosyl- and sialyltransferases. Moreover, the data allows an estimation of the overall contribution of the two heavy chain fused protein domains to the overall averaged charge resulting from the sialic acid content within the total glycan distribution, and the site occupancy at the N1 and N2 sites (SI = 2 × [SI_N1_ × SOI_N1_ × (1-MI1) + SI_N2_ × SOI_N2_ × (1-MI2)] = 6.34). 

When dealing with model proteins as used in this case study, i.e., containing both Fc and non-Fc glycans, the indices may be calculated separately for Fc and non-Fc glycans. For example, when clearance is of particular interest the levels of galactosylation and sialylation in complex-type Fc glycans are less relevant as these glycans are sterically less accessible compared to exposed glycans and, therefore, do not significantly influence the ASGPR-mediated clearance. Thus, in this case study, calculating only the SI and SE at the overall level on the exposed glycans (N1 and N2) would render the index more indicative of a potential impact on clearance. 

In terms of variability, the robustness study revealed relatively low CV values of up to 3.4% for most indices (SI, SE, AI, and cFI). However, as expected, higher CV values were observed (e.g., 10–60%) when indices are calculated on low-abundant glycans, e.g., MI, GI, %O-acetyl, and %Neu5Gc. Even though these individual indices show high CV values, they still prove to be useful for molecular and process fingerprinting. 

To further assess the variability for the reported indices, within the same analytical session, a repeatability study was performed by analyzing two replicates of three independent preparations of the RHS performed by two operators (for details, see Table A1). As expected, as within a given analytical session, lower variabilities were obtained (e.g., CV ≤ 1.2% for the indices SI, SE, AI, and cFI) while higher variabilities (e.g., CV up to 15%) were observed for other indices that were calculated on the glycan species having low absolute peak areas. Nevertheless, they still provide useful information with regards to molecular/process fingerprinting and the assessment of potential risks.

For this model protein, the matrix provides additional relevant information regarding the glycan macro-heterogeneity of the RHS. For example, while both Fc and N1 glycosylation sites are fully occupied (SOI = 1), the N2 is only partially occupied (SOI = 0.6). In other words, with regards to the N2 site, there are two heavy chain populations: population A in which the N2 is glycosylated (60%), and population B in which the N2 is not glycosylated (40%) (see Table 5 for the characterization matrix of the individual populations). 

**Figure 3:**
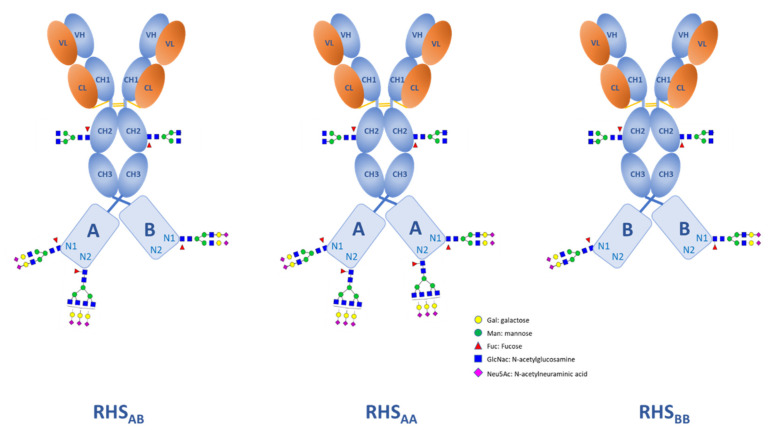
Macro-heterogeneities resulting from the partial site occupancy of the N2 site (ordered according to their expected abundance.

As the overall molecule is composed of two fused heavy chains, the combination results in three distinct RHS population, as illustrated in Figure 3, in which both, one, or none of the N2 glycosylation sites are occupied (populations RHS_AA_, RHS_AB_, and RHS_BB_), respectively. 

Each of the population contributes differently towards the overall characteristics of the molecule, e.g., charge distribution as a function of sialylation: SI_AA_ = (4.14 + 4.14) = 8.28, SI_AB_ = (4.14 + 1.71) = 5.85, and SI_BB_= (1.71 + 1.71) = 3.42. Therefore, the heterogeneity of the overall molecule will also be reflected in the analytical results obtained from other orthogonal analytical techniques such as mass- or charge-based separation techniques. 

The glycan characterization matrix also facilitates risk-based assessments of glycan-related CQAs and their potential impact on efficacy, PK, safety, and immunogenicity. As suggested by Rathore and Malani, such assessments should also focus, besides criticality, on the abundance of relevant glycan features in a molecule [99]. To this end, the glycan characterization matrix provides quantitative metrics for the overall content of glycan-related CQAs, such as sialic acid, Neu5Gc, HM, as well as the sialylation extent of the fused domains. As shown in Table 5, the RHS model protein contains very low amounts of Neu5Gc suggesting a low risk for these glycan-related CQAs.

The glycosylation characterization matrix is also a powerful tool to assess glyco-similarity during process development or in the frame of comparability studies. Table 6 shows a comparison of six candidate processes (P1–P6) based on their glycosylation profile. 

In this scenario, the matrix comparison greatly facilitates the evaluation of the impact of process conditions on the content of individual glycan features. Here, the comparison revealed clear glycosylation differences at site-specific and whole molecule levels in terms of sialylation, antennarity, sialylation extent, high mannose, galactosylation, O-acetylation, and Neu5Gc content demonstrating the powerfulness of the proposed approach as a supporting tool for the selection/decision making of a suitable manufacturing process condition as by the required glycosylation CQAs profile. 

Table 7 illustrates the example of a comparability study to assess product quality after a change in the manufacturing process.

The reported results provide direct evidence of comparable glycosylation profiles, both at the site-specific and at the overall molecule level between the two processes. Therefore, these results indicate that any potential risk regarding immunogenicity (potential impact from Neu5Gc, and HM) or clearance (potential impact from sialic acid content, sialylation extent, Neu5Gc, and HM) remain unchanged with very minor differences. 

### 3.4. Case Study 2: Comparability Study of Three Recombinant FSH Products 

**Figure 4:**
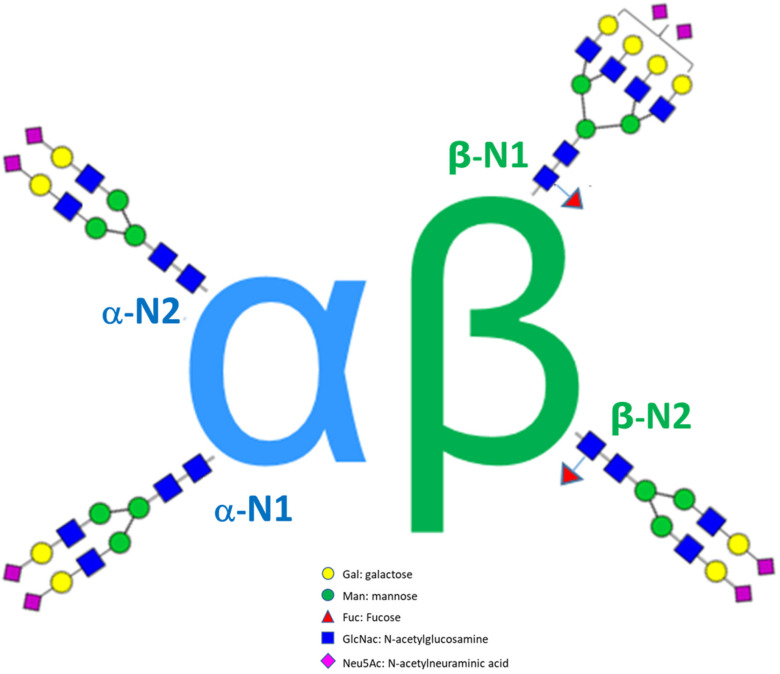
Schematic structure of recombinant FSH.

The recombinant human follicle-stimulating hormone (FSH) is used clinically to induce multiple follicular development in women as part of assisted reproductive technologies. FSH is a heterodimeric molecule consisting of two subunits, a 92-amino acid α subunit and a 111-amino acid β subunit. FSH contains four N-linked glycans, two in the α-subunit (a-N1 and a-N2) and two in the β-subunits (β-N1 and β-N2), with the glycosylation profile of each subunit playing a critical role in the activity and clearance of the whole FSH molecule (Figure 4). 

Table 8 summarizes the glycosylation profiles of three different recombinant FSH products (at a single batch level; more detailed results are reported in Table A2). 

Although the amino acid sequences of the α and β subunits in each product are identical, their glycosylation profiles are product specific representing their respective manufacturing process conditions. The observed glycosylation matrices at the whole molecule level revealed a higher antennarity and a higher sialylation level in product B and a higher N-acetyllactosamine site-specific level at β-N1 in product C. Regarding Neu5Gc, the glycosylation matrix reveals that Product C differs significantly from Products A and B due to its higher Neu5Gc content (approximately 23% of the molecules contain one Neu5Gc residue).

Furthermore, the glycosylation matrix highlights interesting site-specific information regarding FSH glycosylation. For example, similar to pituitary FSH, the a-subunit glycans are almost entirely afucosylated while in the β subunit, the b-N2 and b-N1 sites are fully and partially fucosylated, respectively. Compared with all other sites, the β-N1 glycans feature higher AI and SI indices, while the SE is significantly lower correlating with a higher site-specific GI. Moreover, β-N1 glycans are preferentially modified with N-acetyllactosamine. The effects resulting from these β-N1 glycan features warrant further investigation. The higher antennarity and the higher number of sialic acid residues could be expected to decrease the clearance resulting in an overall positive contribution to the in vivo FSH potency. On the other hand, the lower SE and higher GI could also be expected to increase the ASGPR-mediated clearance with a negative contribution to the in vivo FSH potency. It could be speculated that such site-specific differences play a role in the regulation of the biological activity of human FSH. 

## 4. Discussion

### 4.1. Need for Standardization 

The lack of standardization for the reporting of different glycan attributes complicates comparisons of observed levels or shifts in glycoform distributions, e.g., between different products or literature reports [10]. Standardization is of particular importance in the frame of risk assessments of critical glycan attributes. For example, a recent review highlighted the difficulties of comparing literature data on the content of Neu5Gc as they are usually not reported in a harmonized fashion and instead frequently expressed in different ratios/units such as mol Neu5Gc/mol protein, %_Neu5Gc_ of total sialic acid, %_Neu5Gc_ of total glycans, mol Neu5Gc/gram protein, or %_Neu5Gc_ (*w*/*w*) [43]. Moreover, different indices can be found in the literature which describe the same molecular glycosylation attribute, e.g., the Z number [25] used as a hypothetical metric for the charge contribution through the sialic acid content in glycans, and the sialylation index [10] describing the degree of sialylation of galactosylated species. Although any unit of measurement may be useful to assess product/process consistency in comparability studies, some units may have low practical value when assessing risks regarding efficacy, safety, and clearance.

To address the lack of standardized metrics in glycan analysis and reporting, especially in the assessment of risks associated with glycan-related CQAs, our approach proposes a general method for the comprehensive characterization and visualization of the glycosylation profile of a biopharmaceutical. This method is based on a set of indices which provide qualitative and quantitative information on glycosylation attributes. Content information is provided on a mole-per-mole basis, e.g., the number of groups per glycosylation site or per molecule. Therefore, this approach greatly facilitates comparative studies, i.e., assessing the glyco-similarity in the frame of comparability and biosimilarity studies as well as to monitor changes in the glycosylation profile during process development and optimization. The matrix of glycan indices provides a unique representation for the molecule and can be further used as a guidance for process control in biomanufacturing. 

### 4.2. Risk Assessment 

Products manufactured in murine cell lines can produce considerable levels of problematic glycan attributes, such as the xeno-antigenic glycan αGal and the xeno-autoantigen Neu5Gc. Their presence in a biotherapeutic, combined with the common occurrence of circulating anti-neu5Gc and anti-αGal antibodies in humans, pose a potential immunogenicity, safety, and/or clearance risk [40,100].

When assessing potential risks of glycan-related CQAs, the mole-per-mole information (i.e., the content/molecule of Neu5Gc, aGal, or HM) is more practical. The indices reported here are calculated at site-specific glycosylation level and, when complemented with site occupancy data, an estimation of the glycan-related CQA index at the overall molecular level can be extrapolated. Moreover, these metrics can easily be translated into amounts of glycan-type administered per drug dose and are thus more suited when comparing potential risks associated with different administered products. For example, SI_Neu5Gc_, αGI, and MI may be helpful for the evaluation of the immunogenicity or safety risk and the potential impact on PK. For the latter, additional relevant indices, such as SI and SE should also be considered. 

### 4.3. Adaptability and Versatility

The two presented case studies exemplified how the glycan matrices can be effectively employed as tools to characterize the micro- and macro-heterogeneity of a product in comparability, biosimilarity studies and to support process development. The glycan matrix can be easily adapted by selecting the most relevant glycan attributes for a given product based on its mode of action and its manufacturing process. 

The analytical methods used on the glycan index approach are robust. Compared to other analytical approaches, the proposed analytical workflow of the glycopeptide analysis does not require complex steps of glycan release or labelling. Therefore, the approach is less affected by potential bias through incomplete glycan release.

The proposed glycan indices are intuitive as opposed to previously reported approaches which introduced hypothetical metrics that were difficult to link with actual glycan features of the molecule. For example, the Z-number [25] and the hypothetical antennarity index A-index [26] are not easily conceivable, e.g., in the context of the actual chemico-physical properties of the molecule. Alternatively, the proposed glycosylation metrics SI and AI can easily be conceived as they represent the average number of sialic acid molecules (charges) and antennae, respectively, that are present at a given site or at whole molecule level. The proposed indices are therefore more informative and easier to visualize. 

Furthermore, the correlation of an index with actual glycan features, e.g., SI, SE, and charge distribution, facilitates correlating results with other orthogonal analyses, e.g., isoforms observed in an isoelectric profile. Particularly for cases such as FSH (see Section 3.4), in which there is an intricate balance between site-specific glycan attributes and biopotency, the glycan matrix method provides an intuitive and powerful tool for the characterization and comparison of glycosylation profiles, e.g., pituitary FSH vs. urinary FSH, and recombinant products.

## 5. Conclusions

A novel approach based on numerical indices is proposed for the comprehensive characterization of glycosylation in biotherapeutics. This approach is generally applicable to therapeutic glycoproteins and addresses the need for standardization in glycan data reporting. The granular site-specific information, with the possibility to extrapolate relevant data to the overall molecular level, provides an intuitive tool for the molecular visualization which forms the basis of a risk assessment and a greater understanding of the potential impact of manufacturing process changes on protein glycosylation. 

The analytical approach can easily be integrated within a MAM method to provide a unique product glycan fingerprint and therefore has considerable potential to streamline QC and product release workflows, as well as for process development and control. 

Future optimizations could focus on extending the technique to O-glycosylation, as well as the analysis of non-canonical glycosylation sites. Furthermore, the numerical nature of the indices could facilitate correlating the obtained scores with in vitro and in vivo data of efficacy, clearance, and immunogenicity of a given biotherapeutic.

## 6. Materials and Methods

### 6.1. Materials

Urea, iodoacetamide (IAM), and dithiothreitol (DTT) were purchased from Sigma (St. Louis, MO, USA). Trypsin and Chymotrypsin (sequencing grade) were purchased from Roche Diagnostics (Mannheim, Germany). Tris-(hydroxymethyl)-aminomethane (TRIS), ethylenediaminetetraacetic acid (EDTA), trifluoroacetic acid (TFA), hydrochloric acid (HCl), and liquid chromatography–mass spectrometry (LC-MS)-grade acetonitrile (ACN) were obtained from Merck (Darmstadt, Germany). Guanidine 8 M solution was purchased from Thermo Fisher Scientific (San Jose, CA, USA). All water used in experiments was purified with a Milli-Q system from Merck Millipore (Milford, MA, USA). Amicon Ultracel 3k centrifugal filters were purchased from Merck Millipore (Tullagreen, Carrigtwohill, Ireland) and the Acquity UPLC BEH glycan column 1.7 μm, 2.1 × 150 mm was purchased from Waters (Milford, MA, USA).

Antibody fusion proteins (RHS and process samples) were provided by Merck KGaA (Darmstadt, Germany).

The recombinant FSH product A was provided by Merck KGaA (Darmstadt, Germany), products B and C were purchased from the market. 

### 6.2. Methods

#### 6.2.1. Glycopeptide Mapping

Antibody fusion molecule and recombinant FSH products were processed according to the procedures previously reported in [101,102], respectively.

The relative quantitation was performed using the peptide ion intensity/area of the specific glycopeptides species as a fraction of the sum of all glycopeptide species. The value is provided as a percent within the relative distribution.

#### 6.2.2. Site Occupancy Analysis 

Site occupancy can be determined by using label and label-free methods [97]. A typical label-free LC-MS approach is based on a classical peptide mapping methodology, wherein the molecule is first deglycosylated and then digested to generate peptides. The relative quantitation is performed using the peptide ion intensity/area of the modified peptide (deglycosylated form) as a fraction of the sum of modified peptide (deglycosyated form) and non-glycosylated peptides. Among the deglycosylation enzymes, N-glycosidase F (PNGase F) is used to deglycosylate the N-glycans and converts the asparagine to aspartic acid with a molecular mass difference of 1 dalton [103]. Alternatively, the use of endoglycosidase H enzyme generates the modified peptides containing a single GlcNAc residue [104]. Apart from these, a wide variety of analytical options are available [105]. 

#### 6.2.3. Index Calculation

The calculation of the indices can be organized as site-specific indices and whole molecule/domain indices to provide the respective glycan fingerprint. The whole molecule/domain indices provide a mole/mole protein value that can be translated in the number of specific glycan features present in a whole protein or whole domain. 

##### Site-Specific Indices

*Sialylation index (SI)*

The ponderal average number of all sialic acid molecules present (Neu5Ac, Neu5Gc, O-acetylated, etc.) is obtained by considering the relative percentage (%) and number (x) of sialic acid residues present in each glycoform, excluding high mannose.

$$\mathrm{SI}=\frac{\sum \left(\%\mathrm{AnGmSx}\times x\right)+\sum \left(\%\mathrm{FAnGmSx}\times x\right)}{\sum \left(\%\mathrm{AnGmSx}\right)+\sum \left(\%\mathrm{FAnGmSx}\right)}\;=\frac{\sum \left(\%\mathrm{AnGmSx}\times x\right)+\sum \left(\%\mathrm{FAnGmSx}\times x\right)}{100}$$

- AnGmSx: non-fucosylated glycoforms with n antennae, m galactose residues, and x sialic acid residues (any type) present.
- FAnGmSx: fucosylated glycoforms with n antennae, m galactose residues, and x sialic acid residues (any type) present.
- n can range from 1 to 5.
- m can range from 0 to n.
- x can range from 0 to m.

*Neu5Gc Sialylation Index (SI_Neu5Gc_)*

The ponderal average number of Neu5Gc present is obtained by considering the relative percentage (%) and number (y) of Neu5Gc residues present in each glycoform, excluding high mannose.

$${\mathrm{SI}}_{\mathrm{Neu}5\mathrm{Gc}}=\frac{\sum \left(\%\mathrm{AnGmSy}\times y\right)+\sum \left(\%\mathrm{FAnGmSy}\times y\right)}{\sum \left(\%\mathrm{AnGmSx}\right)+\sum \left(\%\mathrm{FAnGmSx}\right)}\;=\frac{\sum \left(\%\mathrm{AnGmSy}\times y\right)+\sum \left(\%\mathrm{FAnGmSy}\times y\right)}{100}$$

- AnGmSy: non-fucosylated glycoforms with n antennae, m galactose residues, and y Neu5Gc residues present.
- FAnGmSy: fucosylated glycoforms with n antennae, m galactose residues, and y Neu5Gc residues present.
- AnGmSx: non-fucosylated glycoforms with n antennae, m galactose residues, and x sialic acid residues (any type) present.
- FAnGmSx: fucosylated glycoforms with n antennae, m galactose residues, and x sialic acid residues (any type) present.
- n can range from 1 to 5.
- m can range from 0 to n.
- x can range from 0 to m.
- y can range from 0 to m.

Alternatively, when SI and the %_Neu5Gc_ are known, SI_Neu5Gc_ may be calculated as follows:

$${\mathrm{SI}}_{\mathrm{Neu}5\mathrm{Gc}}=\frac{SI\times {\%}_{\mathrm{Neu}5\mathrm{Gc}}}{100}$$

*Sialylation Extent (SE)*

The ponderal average sialylation degree is obtained by considering each glycoform estimated in terms of its relative percentage (%), the number (x) of sialic acid residues present (0–5), and the total number of sialic acid that a given glycoform could potentially accommodate (x_max_, corresponds to the number of antennae present). Therefore, SE represents a measurement of the extent of sialic acid “end-capping” on terminal galactoses of the sugar chains.

$$\mathrm{SE}=\frac{\sum \left(\%\mathrm{AnGmSx}\times \frac{x}{x_{\max}}\right)+\sum \left(\%\mathrm{FAnGmSx}\times \frac{x}{x_{\max}}\right)}{100}$$

- AnGmSx: non-fucosylated glycoforms with n antennae, m galactose residues, and x sialic acid residues (any type) present.
- FAnGmSx: fucosylated glycoforms with n antennae, m galactose residues, and x sialic acid residues (any type) present.
- n can range from 1 to 5.
- m can range from 0 to n.
- x can range from 0 to m.

*SIα2,6*

The ponderal average number of all sialic acids with α2,6 linkages present in the antennae distribution.

$${\mathrm{SI}}_{\alpha 2,6}=\frac{\sum \left(\%\mathrm{AnGmSy}\times y\right)+\sum \left(\%\mathrm{FAnGmSy}\times y\right)}{\sum \left(\%\mathrm{AnGmSx}\right)+\sum \left(\%\mathrm{FAnGmSx}\right)}\;=\frac{\sum \left(\%\mathrm{AnGmSy}\times y\right)+\sum \left(\%\mathrm{FAnGmSy}\times y\right)}{100}$$

- AnGmSy: non-fucosylated glycoforms with n antennae, m galactose residues, and y sialic acid residues in α2,6 linkage present.
- FAnGmSy: fucosylated glycoforms with n antennae, m galactose residues, and y sialic acid residues in α2,6 linkage present.
- AnGmSx: non-fucosylated glycoforms with n antennae, m galactose residues, and x sialic acid residues (any type or linkage) present.
- FAnGmSx: fucosylated glycoforms with n antennae, m galactose residues, and x sialic acid residues (any type or linkage) present.
- n can range from 1 to 5.
- m can range from 0 to n.
- x can range from 0 to m.
- y can range from 0 to m.

Alternatively, when SI and the %_α2,6_ are known, Siα2,6 may be calculated as follows:

$${\mathrm{SI}}_{\alpha 2,6}=\frac{\mathrm{SI}\;\times {\;\%}_{\alpha 2,6}}{100}$$

*Neu5Gc **%***

Relative percent of Neu5Gc within total sialylation.

$${\%}_{\mathrm{Neu}5\mathrm{Gc}}=\frac{{\mathrm{SI}}_{\mathrm{Neu}5\mathrm{Gc}}}{\mathrm{SI}}\times 100\;=\frac{\sum \left(\%\mathrm{AnGmSy}\;\times \;y\right)+\sum \left(\%\mathrm{FAnGmSy}\;\times \;y\right)}{\sum \left(\%\mathrm{AnGmSx}\;\times \;x\right)+\sum \left(\%\mathrm{FAnGmSx}\;\times \;x\right)}\times 100$$

- AnGmSy: non-fucosylated glycoforms with n antennae, m galactose residues, and y Neu5Gc residues present.
- FAnGmSy: fucosylated glycoforms with n antennae, m galactose residues, and y Neu5Gc residues present.
- AnGmSx: non-fucosylated glycoforms with n antennae, m galactose residues, and x sialic acid residues (any type) present.
- FAnGmSx: fucosylated glycoforms with n antennae, m galactose residues, and x sialic acid residues (any type) present.
- n can range from 1 to 5.
- m can range from 0 to n.
- x can range from 0 to m.
- y can range from 0 to m.

*O-acetylated %*

Relative % of O-acetylated sialic acid within total sialylation is obtained by considering the relative percentage (%) and number (y) of O-acetylation present on sialic acid residues.

$$\%{\;}_{O-\mathrm{acetylated}}=\frac{\sum \left(\%\mathrm{AnGmSy}\times y\right)+\sum \left(\%\mathrm{FAnGmSy}\times y\right)}{\sum \left(\%\mathrm{AnGmSx}\times x\right)+\sum \left(\%\mathrm{FAnGmSx}\times x\right)}\times 100$$

- AnGmSy: non-fucosylated glycoforms with n antennae, m galactose residues, and y O-acetylated sialic acid percent residues present.
- FAnGmSy: fucosylated glycoforms with n antennae, m galactose residues, and y O-acetylated sialic acid percent residues present.
- AnGmSx: non-fucosylated glycoforms with n antennae, m galactose residues, and x sialic acid residues (any type) present.
- FAnGmSx: fucosylated glycoforms with n antennae, m galactose residues, and x sialic acid residues (any type) present.
- n can range from 1 to 5.
- m can range from 0 to n.
- x can range from 0 to m.
- y can range from 0 to m.

*A-index (AI)*

The ponderal average number of antennae present is obtained by considering each glycoform estimated in terms of its relative percentage (%) and the number (n) of antennae present.

$$\mathrm{AI}=\frac{\sum \left(\%\mathrm{AnGmSx}\times n\right)+\sum \left(\%\mathrm{FAnGmSx}\times n\right)}{\sum \left(\%\mathrm{AnGmSx}\right)+\sum \left(\%\mathrm{FAnGmSx}\right)}\;=\frac{\sum \left(\%\mathrm{AnGmSx}\times n\right)+\sum \left(\%\mathrm{FAnGmSx}\times n\right)}{100}$$

- AnGmSx: non-fucosylated glycoforms with n antennae, m galactose residues, and x sialic acid residues (any type or linkage) present.
- FAnGmSx: fucosylated glycoforms with n antennae, m galactose residues, and x sialic acid residues (any type or linkage) present.
- n can range from 1 to 5.
- m can range from 0 to n.
- x can range from 0 to m.

*Site occupancy index (SOI)*

SOI is the relative proportion of glycan present in a specific site. SOI = 1.0: the site is fully occupied by the glycan, SOI = 0.0: the site is not occupied. 

The relative quantitation is performed as a percentage using the peptide ion intensity/area of the modified peptide (deglycosylated form) as a fraction of the sum of modified peptide (deglycosylated form) and non-glycosylated peptides.

$$\mathrm{SOI}=\frac{\sum ({\%}_{\mathrm{glycosylated}})}{\sum ({\%}_{\mathrm{glycosylated}})+\sum ({\%}_{\mathrm{non}-\mathrm{glycosylated}})}\;=\frac{\sum ({\%}_{\mathrm{glycosylated}})}{100}$$

*Core Fucosylation index (cFI)*

Relative level of core fucosylated glycan present at a specific site. cFI = 1.0: the glycan present at a given site is fully fucosylated (100%), cFI = 0: the glycan is not fucosylated (0%).

$$\mathrm{cFI}\;=\frac{\sum \left(\%\;\mathrm{FAnGmSx}\right)}{\sum \%\;\mathrm{all}\;\mathrm{species}}\;=\frac{\Sigma \;\left(\%\;\mathrm{FAnGmSx}\right)\;}{100}$$

- FAnGmSx: fucosylated glycoforms with n antennae, m galactose residues, and x sialic acid residues (any type or linkage) present;
- n can range from 1 to 5;
- m can range from 0 to n;
- x can range from 0 to m.

*Antennae fucose Index (aFI)*

Ponderal average number of fucose residues present in the antennae.

$$\alpha \mathrm{FI}\;=\frac{\Sigma \;\left(\%\;\mathrm{aFAnGmSx}\;\times \;\mathrm{yaF}\right)}{\sum \left(\%\;\mathrm{all}\;\mathrm{species}\right)}\;\;=\frac{\Sigma \;\left(\%\;\mathrm{aFAnGmSx}\;\times \;\mathrm{yaF}\right)\;}{100}$$

- yaF: number of fucose residues present in the antennae;
- aFAnGmSx: antennae fucosylated glycoforms with n antennae, m galactose residues, and x sialic acid residues (any type or linkage) present;
- n can range from 1 to 5;
- m can range from 0 to n;
- x can range from 0 to m.

*Galactosylation Index (GI)*

The ponderal average number of terminal galactose residues (excluding high mannose species M5 to M9) within the total glycan distribution.

$$\mathrm{GI}\;=\frac{\Sigma \;\left(\%\;\mathrm{AnGmSx}\;\times m\right)+\;\Sigma \;\left(\%\;\mathrm{FAnGmSx}\;\times m\right)}{100}$$

- m: number of galactose residues present in the glycan.
- AnGmSx: non-fucosylated glycoforms with n antennae, m galactose residues, and x sialic acid residues (any type or linkage) present:
- FAnGmSx: fucosylated glycoforms with n antennae, m galactose residues, and x sialic acid residues (any type or linkage) present.
- n can range from 1 to 5.
- m can range from 0 to n.
- x can range from 0 to m.

*G0 (only for molecule containing the Fc domain)*

The percent complex-type glycans with 0 galactose residues.

$$G0=\frac{\Sigma \;\left(\%\;\mathrm{AnG}0\right)+\;\Sigma \;\left(\%\;\mathrm{FAnG}0\right)}{\sum \left(\%\;\mathrm{all}\;\mathrm{species}\right)}\times 100$$

- AnG0: non-fucosylated glycoforms with n antennae and 0 galactose residues;
- FAnG0: fucosylated glycoforms with n antennae and 0 galactose residues.

*G1_1,6_ (only for molecules containing the Fc domain)*

The percent complex-type glycans with the isomeric galactose residue on the α1,6 arm of the oligosaccharide.

$$G1_{1,6}=\frac{\sum \left(\%\mathrm{AnG}1_{1,6}\right)+\sum \left(\%\mathrm{FAnG}1_{1,6}\right)}{\sum \left(\%\;\mathrm{all}\;\mathrm{species}\right)}\times 100$$

- AnG1_1,6_: non-fucosylated glycoforms with n antennae and 1 galactose residue on the α1-6 arm:
- FAnG1_1,6_: fucosylated glycoforms with n antennae and 1 galactose residue on the α1,6 arm;
- Note: the distinction between G1_1,6_ and G1_1,3_ is only possible with dedicated analytical techniques.

*G2 (only for molecules containing the Fc domain)*

The percent complex-type glycans with 2 galactose residues (fully galactosylated).

$$G2=\frac{\sum \left(\%\mathrm{AnG}2\right)+\sum \left(\%\mathrm{FAnG}2\right)\;}{\sum \left(\%\;\mathrm{all}\;\mathrm{species}\right)}\times 100$$

- AnG2: non-fucosylated glycoforms with n antennae and 2 galactose residues.
- FAnG2: fucosylated glycoforms with n antennae and 2 galactose residues.
- n can range from 0 to 2.

*α Gal Index (αGI)*

The ponderal average number of galactose residues in the alpha-1-3 linkage present in each glycoform, excluding high mannose.

$$\alpha \mathrm{GI}\;=\frac{\sum \left(\%\;\mathrm{AnGmSx}\;\times \;y\alpha G\right)+\sum \left(\%\;\mathrm{FAnGmSx}\;\times \;y\alpha G\right)}{\sum \left(\%\;\mathrm{all}\;\mathrm{species}\right)}$$

- yαG: number of galactose residues in alpha-1-3 linkage.
- AnGmSx: non-fucosylated glycoforms with n antennae, m galactose residues, and x sialic acid residues (any type or linkage) present.
- FAnGmSx: fucosylated glycoforms with n antennae, m galactose residues, and x sialic acid residues (any type or linkage) present.
- n can range from 1 to 5.
- m can range from 0 to n.
- x can range from 0 to m.

*N-acetyllactosamine (LacNAc) Index (LI)*

The ponderal average number of LacNAc (Gal-GlcNAc) units within the total glycan distribution.

$$\mathrm{LI}\;=\frac{\sum \left(\%\;\mathrm{AnGmSx}\;\times \;\mathrm{yLacNAc}\right)+\sum \left(\%\;\mathrm{FAnGmSx}\;\times \;\mathrm{yLacNAc}\right)\;}{100}$$

- yLacNAc: number of N-acetyllactosamine (LacNAc) units present in the glycan.
- AnGmSx: non-fucosylated glycoforms with n antennae, m galactose residues, and x sialic acid residues (any type or linkage) present.
- FAnGmSx: fucosylated glycoforms with n antennae, m galactose residues, and x sialic acid residues (any type or linkage) present.
- n can range from 1 to 5.
- m can range from 0 to n.
- x can range from 0 to m.

*Mannose Index (MI)*

The ponderal average number of high mannose structures (M5–M9) within the total glycan distribution.

$$\mathrm{MI}=\frac{\sum \left(\%\mathrm{Mm}\right)}{100}$$

- m: number of mannose residues present in the high mannose structures (M5–M9).
- m can range from 5 to 9;
- Note: the MI only takes into consideration the high mannose structures (M5–M9) in the calculation.

*Hybrid Index (HI)*

The ponderal average number of hybrid structures within the total glycan distribution.

$$\mathrm{HI}=\frac{\sum \left(\%\mathrm{Hybrid}\right)}{100}$$

*Bisecting Index (BI)*

The ponderal average number of bisecting structures within the total glycan distribution.

$$\mathrm{BI}=\frac{\sum \left(\%\mathrm{Bisecting}\right)}{100}$$

##### Calculation of the Indices for the Whole Molecule/Domains

Most of the above-mentioned indices are calculated on the basis of complex-type glycans, i.e., excluding high-mannose type glycans. For the calculation of the corresponding indices for the whole molecule or for individual domains, the conversion factor (1-MI) is applied, which ensures that the entire glycan population, i.e., including high-mannose type glycans, is taken into consideration. Thus, whole molecule/domain-specific indices are calculated by adding together the individual site-specific indices each multiplied with their respective SOI and their respective conversion factor (1-MI). For molecules containing homomultimeric chains, e.g., antibody fusion molecules, the number of glycosylated chains, e.g., 2 heavy chains, must be taken into consideration in the index calculation for the whole molecule. These calculations are required in order to obtain more representative indices in terms of the ponderal average mole/mole _protein_ values.

SI=∑(SIi×SOIi×n×(1-MIi)) 

AI=∑(AIi×SOIi×n×(1-MIi)) 

$$\mathrm{SE}=\frac{\mathrm{SI}}{\mathrm{AI}}$$

$${\mathrm{SI}}_{\mathrm{Neu}5\mathrm{Gc}}=\sum ({\mathrm{SI}}_{\mathrm{Neu}5\mathrm{Gc}}\times \mathrm{SOIi}\times n\times (1-\mathrm{MIi}))\;$$

αGI=∑(αGIi×SOIi×n×(1-MIi)) 

MI=∑(MIi×SOIi×n) 

LI=∑(LIi×SOIi×n) 

aFI=∑(aFIi×SOIi×n×(1-MIi))

where i is the site index, (1-MIi) is the conversion factor that considers the high mannose content, and n is the number of homomultimeric chains containing the glycosylation site. 

## 7. Patents

International Publication number WO 2022/200262 A1 titled: ‘Method for glycosylation profiling to describe functional characteristics of a biologic molecule’ is related to the work reported in the manuscript.

## Figures and Tables

**Table 1:** Main N-glycan-related indices to be considered at site-specific and whole-molecule level for different classes of biotherapeutics produced by different expression systems.

Cell Line	Product Class	Glycan Related Indices Per Site																				Whole Molecule Glycosylation Indices						
		SOI	AI	SI	SI_α2,6_	SE	SI_Neu5Gc_	% _Neu5Gc_	% _O-acetyl_	αGI	GI	G2	G1_1-6_	G1	G0	MI	HI	cFI	aFI	LI	BI	AI	SI	SE	SI_α2,6_	SI-_Neu5Gc_	αGI	MI
Murine	mAbs (only Fc glycosylation)	✓	✓	✓	✓	✓	✓	✓		✓	✓	✓_Fc_	✓ _Fc_	✓_Fc_	✓_Fc_	✓	✓	✓			✓					✓	✓	✓
	mAbs with Fc and Fab glycosylation	✓	✓	✓	✓	✓	✓	✓	✓	✓	✓	✓ _Fc_	✓ _Fc_	✓ _Fc_	✓ _Fc_	✓	✓	✓	✓	✓	✓	✓	✓	✓	✓	✓	✓	✓
	Fc and antibody fusion proteins	✓	✓	✓	✓	✓	✓	✓	✓	✓	✓	✓ _Fc_	✓ _Fc_	✓ _Fc_	✓ _Fc_	✓	✓	✓	✓	✓	✓	✓	✓	✓	✓	✓	✓	✓
	Any other class of protein	✓	✓	✓	✓	✓	✓	✓	✓	✓	✓					✓	✓	✓	✓	✓	✓	✓	✓	✓	✓	✓	✓	✓
CHO	Mab (only Fc glycosylation)	✓	✓	✓		✓	✓	✓	✓	✓	✓	✓ _Fc_	✓ _Fc_	✓ _Fc_	✓ _Fc_	✓	✓	✓								✓	✓	✓
	mAbs with Fc and Fab glycosylation	✓	✓	✓		✓	✓	✓	✓	✓	✓	✓ _Fc_	✓ _Fc_	✓ _Fc_	✓ _Fc_	✓	✓	✓	✓	✓	✓	✓	✓	✓		✓	✓	✓
	Fc and antibody fusion proteins	✓	✓	✓		✓	✓	✓	✓	✓	✓	✓ _Fc_	✓ _Fc_	✓ _Fc_	✓ _Fc_	✓	✓	✓	✓	✓	✓	✓	✓	✓		✓	✓	✓
	Any other class of protein	✓	✓	✓		✓	✓	✓	✓	✓	✓					✓	✓	✓	✓	✓	✓	✓	✓	✓		✓	✓	✓
Human	Mab (only Fc glycosylation)	✓	✓	✓	✓	✓					✓	✓ _Fc_	✓ _Fc_	✓ _Fc_	✓ _Fc_	✓	✓	✓			✓							✓
	mAbs with Fc and Fab glycosylation	✓	✓	✓	✓	✓			✓		✓	✓ _Fc_	✓ _Fc_	✓ _Fc_	✓ _Fc_	✓	✓	✓	✓	✓	✓	✓	✓	✓	✓			✓
	Fc and antibody fusion proteins	✓	✓	✓	✓	✓			✓		✓	✓ _Fc_	✓ _Fc_	✓ _Fc_	✓ _Fc_	✓	✓	✓	✓	✓	✓	✓	✓	✓	✓			✓
	Any other class of protein	✓	✓	✓	✓	✓			✓		✓					✓	✓	✓	✓	✓	✓	✓	✓	✓	✓			✓

The tick marks indicate the glycan indices recommended for different classes of biopharmaceuticals expressed in commonly used expression systems. **SOI**: site occupancy index—relative level of glycan present in a specific site. SOI = 1: the site is fully occupied (100%) by the glycan, SOI = 0: not occupied (0%); **AI**: antennarity index—ponderal average number of antennae present and is obtained considering each glycoform estimated in terms of its relative percentage (%) and the number (n) of antennae present; **SI**: sialylation index—ponderal average number of all sialic acid residues present (Neu5Ac + Neu5Gc + O-acetylated) on complex-type and hybrid-type glycans; **SI_α2,6_**: sialylation index of sialic acid in α2,6 linkage—ponderal average number of all sialic acid residues (on complex- and hybrid-type glycans) with α2,6 linkage; **SE**: sialylation extent—ponderal average of the extent of sialic acid “end-capping” on terminal galactoses (on complex- and hybrid-type glycans) as a proportion of the total number of antennae present in the glycans; **SI_Neu5Gc_**: Neu5Gc sialylation index—ponderal average number of Neu5Gc residues present on complex-type and hybrid-type glycans; **%Neu5Gc**: percentage of Neu5Gc within total sialylation; **%O-acetylated**: percentage of O-acetylated sialic acid within total sialylation; **αGI**: αGal index—ponderal average number of galactose residues in an alpha-1-3 linkage (complex- and hybrid-type glycans); **GI**: galactosylation index—ponderal average number of terminal galactose residues within the glycan distribution of complex- and hybrid-type glycans; **G0**: percentage of Fc-glycans (complex-type) with zero galactose residues present on the Fc-oligosaccharides; **G1**: percentage of Fc-glycans (complex-type) with one galactose residue on the Fc-oligosaccharide on either the α1-6 arm or the α1-3 arm; **G1_1-6_**: percentage of Fc-glycans (complex-type) with the isomeric galactose residue on the α1-6 arm of the Fc-oligosaccharide; **G2**: percentage of Fc-glycans (complex-type) with two terminal galactose residues (fully galactosylated Fc-glycan); **MI**: high mannose index—ponderal average number of high mannose structures (M5–M9) present; **HI:** Hybrid-type glycan index—relative level of hybrid structures within the total glycan distribution; **cFI**: core fucose index—relative level of core fucosylation present at a given site; **aFI**: antennae fucose index—ponderal average number of fucose residues present in the antennae; **LI**: N-acetyllactosamine index—ponderal average number of LacNAc (Gal-GlcNAc) units present (complex- and hybrid-type glycans); **BI**: bisecting index—relative level of bisecting structures within the total complex-type glycan distribution.

**Table 2:** Cell-line-specific glycan-related PTMs.

Cell Line	Specific PTM	Potential Impact
**Murine** **(NS0 and Sp2/0)**	αGal	Immunogenicity, clearance, and safety
	Neu5Gc	Immunogenicity, clearance, and safety
	Sialic acid linkage α2,3 and α2,6	Impact on clearance dependent on SE and SI. In rodents, glycoproteins carrying α2,6-linked sialic acid, but not those carrying α2,3-linked sialic acid, have been reported to interact with the asialoglycoprotein receptor resulting in a more rapid clearance [27]. A comparable effect in humans is still to be demonstrated. However, a differential impact on clearance cannot be ruled out.
**CHO**	Neu5Gc (low level)	Immunogenicity, clearance, and safety
	αGal (possible very low level)	Immunogenicity, clearance, and safety
	Sialic acid linkage: only α2,3	Impact on clearance dependent on SE and SI.
**Human**	Sialic acid linkage α2,3 and α2,6	Impact on clearance dependent on SE and SI. In rodents, glycoproteins carrying α2,6-linked sialic acid, but not those carrying α2,3-linked sialic acid, have been reported to interact with the asialoglycoprotein receptor resulting in a more rapid clearance [27]. A comparable effect in humans is still to be demonstrated. However, a differential impact on clearance cannot be ruled out.
	Bisecting GlcNAc	Impedes core fucosylation. Therefore, has an impact on mAb efficacy in Fc glycans. Potential interaction with C-type lectin receptors in exposed glycans.

**Table 3:** An example matrix of the N-glycan-related indices, relevant at site-specific and whole molecule level, for an antibody Fc fusion protein, expressed in CHO cells, as exemplified in Figure 2.

	Glycan Related CQA Indices																			
Glycosite	SOI	AI	SI	SI_α2,6_	SE	SI _Neu5Gc_	% _Neu5Gc_	% _O-acetyl_	αGI	GI	G2	G1_1-6_	G1	G0	MI	HI	cFI	aFI	LI	BI
Fc	✓	✓	✓	NA	✓	✓	✓	NA	✓	✓	✓	✓	✓	✓	✓	✓	✓	NA	NA	NA
N1	✓	✓	✓	NA	✓	✓	✓	✓	✓	✓	NA	NA	NA	NA	✓	✓	✓	✓	✓	NA
N2	✓	✓	✓	NA	✓	✓	✓	✓	✓	✓	NA	NA	NA	NA	✓	✓	✓	✓	✓	NA
Whole molecules/domains	NA	✓	✓	NA	✓ *	✓	NA	NA	✓	NA	NA	NA	NA	NA	✓	NA	NA	✓	✓	NA

The tick marks indicate the recommended glycan indices. NA: Not Applicable; *: Whole SE is calculated from the ratio whole SI/whole AI.

**Table 4:** RHS glycan characterization matrix.

Glycosite		RHS Characterization Matrix									
		SOI	AI	SI	SE	SI_Neu5Gc_	%_Neu5Gc_	%_O-acetyl_	GI	MI	cFI
Fc	Mean	1.0	1.77	ND	<0.01	0.00	ND	ND	0.20	0.02	0.98
	CV%	- ^(1)^	1.54	NA	NA	NA	NA	NA	10.69	23.99	0.52
	StDev	- ^(1)^	0.03	NA	NA	0.00	NA	NA	0.02	0.00	0.01
N1	Mean	1.0	2.64	1.73	0.65	0.01	0.34	2.29	0.64	0.01	0.99
	CV%	- ^(1)^	2.50	3.39	2.85	30.68	29.50	56.98	7.20	15.74	0.32
	StDev	- ^(1)^	0.07	0.06	0.02	0.00	0.10	1.30	0.05	0.00	0.00
N2	Mean	0.6	3.42	2.43	0.71	0.00	0.20	1.96	0.80	ND	1.00
	CV%	- ^(1)^	0.78	1.33	1.45	0.00	26.41	59.55	3.93	NA	0.46
	StDev	- ^(1)^	0.03	0.03	0.01	25.64	0.05	1.17	0.03	NA	0.00
Overall whole molecule/domains			9.33	6.34	0.68	0.02				0.06	

^(1)^—analysis only on one batch. ND—not determined; NA—not applicable; CV—coefficient of variation; StDev—standard deviation.

**Table 5:** Scenario of the heavy chain fused protein domain populations (A, SOI_N2_ = 1) and (B, SOI_N2_ = 0) present in the RHS molecule.

**A**	**60% Fused Protein Domain with N1 and N2 Glycosylated Sites**									
Glycosite	SOI	AI	SI	SE	SI_Neu5Gc_	%_Neu5Gc_	%_O-acetyl_	GI	MI	cFI
Fc	1.0	1.77	ND	<0.01	ND	ND	ND	0.20	0.02	0.98
N1	1.0	2.64	1.73	0.65	0.01	0.34	2.29	0.64	0.01	0.99
N2	1.0	3.42	2.43	0.71	0.00	0.20	1.96	0.80	0.00	1.00
Fused domain A		6.03	4.14	0.69	0.01				0.03	
**B**	**40% Fused Domain with N1 Glycosylated Sites Only**									
Glycosite	SOI	AI	SI	SE	SI_Neu5Gc_	%_Neu5Gc_	%_O-acetyl_	GI	MI	cFI
Fc	1.0	1.77	ND	<0.01	ND	ND	ND	0.20	0.02	0.98
N1	1.0	2.64	1.73	0.66	0.01	0.34	2.29	0.64	0.01	0.99
N2	0.0	0.00	0.00	0.00	0.00	0.00	0.00	0.00	0.00	0.00
Fused domain B		2.61	1.71	0.66	0.01				0.03	

**Table 6:** Modulation of glycan-related CQAs during Process Optimization. P1–P6 represent different process conditions. Indices considered relevant for the whole molecule/domains are calculated and shown in the table.

	N-Glycan Sites	RHS	P1	P2	P3	P4	P5	P6
AI	Fc	1.81	1.82	1.76	1.72	1.82	1.88	1.77
	N1	2.59	2.53	2.54	2.50	2.53	2.61	2.57
	N2	3.46	3.41	3.58	3.50	3.39	3.27	3.54
	Whole	9.28	9.10	9.33	9.15	9.08	9.09	9.39
SI	Fc	ND	ND	ND	ND	ND	ND	ND
	N1	1.68	1.43	2.08	2.04	1.45	2.14	1.47
	N2	2.47	2.38	3.02	3.04	2.38	2.21	3.03
	Whole	6.29	5.69	7.74	7.69	5.73	6.89	6.58
SE	Fc	ND	ND	ND	ND	ND	ND	ND
	N1	0.64	0.56	0.82	0.82	0.57	0.83	0.56
	N2	0.71	0.68	0.85	0.88	0.68	0.66	0.86
	Whole	0.68	0.62	0.83	0.84	0.63	0.76	0.70
SI_Neu5Gc_	Fc	ND	ND	ND	ND	ND	ND	ND
	N1	0.01	0.01	0.03	0.03	0.01	0.02	0.02
	N2	0.00	0.01	0.03	0.02	0.01	0.02	0.04
	Whole	0.02	0.04	0.10	0.10	0.04	0.07	0.08
%_Neu5Gc_	Fc	ND	ND	ND	ND	ND	ND	ND
	N1	0.30	0.71	1.62	1.70	0.98	0.94	1.10
	N2	0.20	0.55	0.99	0.79	0.59	1.13	1.19
%_O-acetyl_	Fc	ND	ND	ND	ND	ND	ND	ND
	N1	1.67	0.21	0.49	0.65	0.21	0.33	0.21
	N2	2.55	0.17	0.33	0.36	0.38	0.18	0.23
GI	Fc	0.17	0.19	0.27	0.30	0.20	0.13	0.22
	N1	0.64	0.30	0.41	0.39	0.28	0.37	0.23
	N2	0.82	0.35	0.51	0.43	0.32	0.26	0.47
MI	Fc	0.01	0.01	0.00	0.00	0.01	0.00	0.00
	N1	0.01	0.01	0.01	0.01	0.01	0.01	0.00
	N2	0.00	0.00	0.00	0.00	0.00	0.00	0.00
	Whole *	0.03	0.03	0.03	0.03	0.02	0.02	0.01
cFI	Fc	0.99	0.99	0.99	0.99	0.99	0.99	0.99
	N1	0.99	0.99	0.99	0.99	1.00	0.99	1.00
	N2	1.00	1.00	1.00	1.00	1.00	1.00	1.00

* Indicated values have an influence on the digit rounding approximations.

**Table 7:** Glycosylation comparability matrix after a process change: two process conditions, pre-change and post-change, were compared by analyzing four batches per process condition, and samples were analyzed in the same analytical session. The table summarizes the average values and variation coefficients of selected glycosylation indices.

	**Pre-Change Condition (Batches n = 4)**									
Glycosite	SOI	AI	SI	SE	SI_Neu5Gc_	%_Neu5Gc_	%_O-acetyl_	GI	MI	cFI
Fc	1.0 ^(1)^	1.74	ND	<0.01	ND	ND	ND	0.24	0.02	0.97
(CV%)		−0.7	NA	NA	NA	NA	NA	−5.9	−14.1	0
N1	1.0 ^(1)^	2.55	1.85	0.72	0.01	0.53	4.09	0.48	0.01	0.99
(CV%)		−1.1	−1.8	−1	−12.9	−11.6	−16.8	−8.2	−22.3	0
N2	0.6 ^(1)^	3.42	2.66	0.77	0.01	0.22	3.97	0.57	ND	1
(CV%)		−0.8	−2.4	−2.1	−16.7	−15.4	−13.8	−8.7	NA	0
2 fused domains		9.15	6.86	0.75	0.03				0.05 *	
	**Post-Change Condition (Batches n = 4)**									
Glycosite	SOI	AI	SI	SE	SI_Neu5Gc_	%_Neu5Gc_	%_O-acetyl_	GI	MI	cFI
Fc	1.0 ^(1)^	1.73	ND	<0.01	ND	ND	ND	0.24	0.02	0.97
(CV%)		−0.6	NA	0	NA	NA	NA	−2.5	−2.5	0
N1	1.0 ^(1)^	2.56	1.91	0.75	0.01	0.46	4.67	0.43	0.01	1
(CV%)		−1	−1.8	−3	−10.9	−12.2	−9	−9.9	−22.3	−0.5
N2	0.6 ^(1)^	3.44	2.66	0.77	0.01	0.22	3.35	0.58	ND	1
(CV%)		−0.8	−1.5	−1.8	−16.7	−17.2	−7.9	−5.7	NA	0
2 fused domains		9.2	6.97	0.76	0.03				0.05 *	

ND: not detected; NA: not applicable; CV: coefficient of variation; ^(1)^—assuming SOI from previous RHS measurements. *—calculated combining the 2 fused domains and the Fc domain.

**Table 8:** FSH comparative matrix of three different FSH products. Averaged values of the different batches tested.

	**Product A (Batches n = 3)**										
Glycosite	SOI	AI	SI	SE	SI_Neu5Gc_	%_Neu5Gc_	%_O-acetyl_	GI	MI	cFI	LI
α-N1	1 ^*1^	2.12	1.75	0.82	0	0.21	2.46	0.37	ND	0.02	0
α-N2	1 ^*1^	2.08	1.77	0.85	ND	ND	4.93	0.32	ND	0.02	0
β-N1	1 ^*1^	3.54	2.25	0.69	ND	ND	2.01	1.43	ND	0.57	0.32
β-N2	1 ^*1^	2.16	1.87	0.88	0	0.18	6.98	0.21	ND	1.02	0
Whole		9.9	7.64	0.77	0.01						0.32
	**Product B (Batches n = 2)**										
Glycosite	SOI	AI	SI	SE	SI_Neu5Gc_	%_Neu5Gc_	%_O-acetyl_	GI	MI	cFI	LI
α-N1	1 ^*1^	2.27	1.79	0.79	0	0.17	0	0.49	ND	0.01	0
α-N2	1 ^*1^	2.24	1.78	0.81	0.01	0.27	0.35	0.47	ND	0.01	0
β-N1	1 ^*1^	4.14	2.7	0.69	ND	ND	0.26	1.22	ND	0.55	0.38
β-N2	1 ^*1^	2.25	1.95	0.88	0.01	0.26	1.05	0.21	ND	1.01	0.01
Whole		10.89	8.2	0.75	0.01						0.39
	**Product C (Batches n = 3)**										
Glycosite	SOI	AI	SI	SE	SI_Neu5Gc_	%_Neu5Gc_	%_O-acetyl_	GI	MI	cFI	LI
α-N1	1 ^*1^	2.1	1.81	0.86	0.08	4.18	1.78	0.28	ND	0.01	0
α-N2	1 ^*1^	2.08	1.86	0.9	0.08	4.31	5.79	0.21	ND	0.01	0
β-N1	1 ^*1^	3.77	2.19	0.64	ND	ND	2.03	1.73	ND	0.49	0.41
β-N2	1 ^*1^	2.22	1.99	0.92	0.07	3.6	6.25	0.11	ND	0.99	0
Whole		10.16	7.85	0.77	0.23						0.41

1 ^*1^—a site occupancy of 1 is assumed (no analytical results); ND—not determined.

## Data Availability

Not applicable.

## Appendix

**Table A1:** Repeatability study applied to RHS (2 operators, 3 independent preparations, 2 replicates per preparation; n = (12)) samples analyzed in the same analytical session.

		RHS Characterization Matrix									
Glycosite		SOI	AI	SI	SE	SI_Neu5Gc_	% _Neu5Gc_	% _O-acetyl_	GI	MI	cFI
Fc	Mean	1.0	1.74	ND	ND	ND	ND	ND	0.24	0.02	1.00
	CV%	- ^(1)^	0.36	NA	NA	NA	NA	NA	1.73	5.42	0.15
	StDev	- ^(1)^	0.01	NA	NA	NA	NA	NA	0.00	0.00	0.00
N1	Mean	1.0	2.64	1.72	0.64	0.01	0.46	3.00	0.64	0.01	1.00
	CV%	- ^(1)^	0.93	1.20	0.66	9.16	9.37	7.16	2.33	10.69	0.21
	StDev	- ^(1)^	0.02	0.02	0.00	0.00	0.04	0.21	0.01	0.00	0.00
N2	Mean	0.6	3.43	2.44	0.71	0.01	0.22	2.18	0.79	ND	1.00
	CV%	- ^(1)^	0.28	0.54	0.41	12.34	12.41	6.93	1.10	NA	0.12
	StDev	- ^(1)^	0.01	0.01	0.00	0.00	0.03	0.15	0.01	NA	0.00
whole molecule/domains			9.34 *	6.33 *	0.68 *	0.03 *				0.05 **	

ND = not detected; NA = not applicable, CV = coefficient of variation, StDev = standard deviation; * = calculated only for the fused protein domain; ** = calculated for the whole molecule. ^(1)^—analysis only on one batch.

**Table A2:** FSH comparative matrix of three recombinant FSH products (Product A and C: three batches tested; Product B: two batches tested).

		Product A			Product B		Product C		
Index	Glycosite	Batch 1	Batch 2	Batch 3	Batch 1	Batch 2	Batch 1	Batch 2	Batch 3
AI	α-N_1_	2.12	2.11	2.12	2.25	2.29	2.08	2.11	2.11
	α-N_2_	2.08	2.07	2.10	2.23	2.25	2.07	2.08	2.08
	β-N_1_	3.60	3.46	3.56	4.15	4.12	3.79	3.85	3.66
	β-N_2_	2.15	2.18	2.16	2.24	2.25	2.20	2.18	2.27
	Whole	9.95	9.82	9.94	10.87	10.91	10.14	10.22	10.12
SI	α-N_1_	1.75	1.74	1.76	1.78	1.79	1.81	1.84	1.79
	α-N_2_	1.76	1.76	1.78	1.77	1.78	1.85	1.87	1.86
	β-N_1_	2.11	2.35	2.29	2.65	2.74	2.17	2.18	2.22
	β-N_2_	1.86	1.88	1.88	1.95	1.94	1.98	2.02	1.97
	Whole	7.48	7.73	7.71	8.15	8.25	7.81	7.91	7.84
SE	α-N_1_	0.83	0.82	0.82	0.80	0.79	0.87	0.87	0.85
	α-N_2_	0.85	0.85	0.86	0.81	0.81	0.90	0.91	0.90
	β-N_1_	0.64	0.73	0.69	0.68	0.70	0.63	0.62	0.66
	β-N_2_	0.88	0.88	0.88	0.88	0.88	0.92	0.94	0.90
	Whole	0.75	0.79	0.78	0.75	0.76	0.77	0.77	0.77
SI_Neu5Gc_	α-N_1_	0.01	0.01	0.00	0.00	0.00	0.07	0.07	0.08
	α-N_2_	0.00	0.00	0.00	0.01	0.01	0.09	0.07	0.08
	β-N_1_	0.00	0.00	0.00	0.00	0.00	0.00	0.00	0.00
	β-N_2_	0.00	0.01	0.00	0.01	0.01	0.07	0.08	0.07
	Whole	0.01	0.01	0.00	0.01	0.01	0.22	0.22	0.24
%_Neu5Gc_	α-N_1_	0.29	0.35	0.00	0.23	0.11	3.87	3.97	4.71
	α-N_2_	0.00	0.00	0.00	0.27	0.27	4.57	3.93	4.43
	β-N_1_	0.00	0.00	0.00	0.00	0.00	0.00	0.00	0.00
	β-N_2_	0.00	0.43	0.11	0.26	0.25	3.48	3.69	3.62
%_O-acetyl_	α-N_1_	2.40	2.59	2.39	0.00	0.00	2.21	2.01	1.12
	α-N_2_	5.51	4.73	4.54	0.38	0.32	5.59	6.54	5.23
	β-N_1_	2.51	2.12	1.40	0.34	0.18	1.66	3.03	1.39
	β-N_2_	7.58	6.24	7.12	1.28	0.81	6.05	7.00	5.71
GI	α-N_1_	0.36	0.37	0.37	0.47	0.50	0.26	0.27	0.31
	α-N_2_	0.32	0.32	0.31	0.46	0.47	0.22	0.20	0.22
	β-N_1_	1.68	1.20	1.41	1.27	1.16	1.79	1.80	1.61
	β-N_2_	0.22	0.20	0.20	0.20	0.21	0.10	0.11	0.12
MI	α-N_1_	ND	ND	ND	ND	ND	ND	ND	ND
	α-N_2_	ND	ND	ND	ND	ND	ND	ND	ND
	β-N_1_	ND	ND	ND	ND	ND	ND	ND	ND
	β-N_2_	ND	ND	ND	ND	ND	ND	ND	ND
	Whole	ND	ND	ND	ND	ND	ND	ND	ND
cFI	α-N_1_	0.02	0.02	0.02	0.01	0.01	0.01	0.01	0.01
	α-N_2_	0.02	0.02	0.02	0.01	0.01	0.01	0.01	0.01
	β-N_1_	0.51	0.63	0.56	0.53	0.56	0.48	0.48	0.51
	β-N_2_	1.02	1.02	1.02	1.01	1.01	0.98	0.99	1.00
LI	α-N_1_	0.00	0.00	0.00	0.00	0.00	0.00	0.00	0.00
	α-N_2_	0.00	0.00	0.00	0.00	0.00	0.00	0.00	0.00
	β-N_1_	0.37	0.26	0.32	0.39	0.37	0.42	0.44	0.36
	β-N_2_	0.00	0.00	0.00	0.01	0.01	0.00	0.00	0.00
	Whole	0.37	0.26	0.32	0.40	0.38	0.42	0.44	0.36
